# Spa therapy together with supervised self-mobilisation improves pain, function and quality of life in patients with chronic shoulder pain: a single-blind randomised controlled trial

**DOI:** 10.1007/s00484-018-1502-x

**Published:** 2018-02-03

**Authors:** Isabelle Chary-Valckenaere, Damien Loeuille, Nicolas Jay, François Kohler, Jean-Noë Tamisier, Christian-François Roques, Michel Boulange, Gérard Gay

**Affiliations:** 1 Rheumatology Department, Nancy University Hospital, and UMR 7365 CNRS-UL IMoPA (Ingéniérie Moléculaire & Physiopathologie Articulaire), Université de Lorraine, 54511 Vandoeuvre-les-Nancy cedex, France; 2 Service Epidémiologie et Statistiques, Nancy University Hospital, Université de Lorraine, 54511 Vandoeuvre-les-Nancy cedex, France; 3 Balneotherapy Care facility, 57360 Paris, France; 4 Paul Sabatier University (Toulouse) and Académie Nationale de Médecine, Paris, France; 5 Hydrologie et Climatologie Médicale, Nancy University Hospital, Université de Lorraine, 54511 Vandoeuvre-les-Nancy cedex, France

**Keywords:** Chronic shoulder pain, Spa therapy, DASH score, SF-36, Balneology, Rotator cuff tendinopathy

## Abstract

To determine whether spa therapy has a beneficial effect on pain and disability in patients with chronic shoulder pain, this single-blind randomised controlled clinical trial included patients with chronic shoulder pain due to miscellaneous conditions attending one of four spa centres as outpatients. Patients were randomised into two groups: spa therapy (18 days of standardised treatment combining thermal therapy together with supervised mobilisation in a thermal pool) and controls (spa therapy delayed for 6 months: ‘immediate versus delayed treatment’ paradigm). All patients continued usual treatments during the 6-month follow-up period. The main endpoint was the mean change in the French-Quick DASH (F-QD) score at 6 months. The effect size of spa therapy was calculated, and the proportion of patients reaching minimal clinically important improvement (MCII) was compared. Secondary endpoints were the mean change in SF-36, treatment use and tolerance. One hundred eighty-six patients were included (94 patients as controls, 92 in the spa group) and analysed by intention to treat. At 6 months, the mean change in the F-QD score was statistically significantly greater among spa therapy patients than controls (− 32.6 versus − 8.15%; *p* < 0.001) with an effect size of 1.32 (95%CI: 0.97–1.68). A significantly greater proportion of spa therapy patients reached MCII (59.3 versus 17.9%). Spa therapy was well tolerated with a significant impact on SF-36 components but not on drug intake. Spa therapy provided a statistically significant benefit on pain, function and quality of life in patients with chronic shoulder pain after 6 months compared with usual care.

## Introduction

Chronic painful shoulder disorders are a heavy burden for society due to health product consumption, sick leave and other vocational and social consequences. After back and neck pain, painful shoulder disorders are the most common musculoskeletal painful condition, needing medical intervention and care (Rekola et al. 1993; Urwin et al. 1998; Linsell et al. 2006). The lifetime prevalence is about one third of the population (Van der Heijden 1999), and community surveys showed a prevalence of between 21 and 34% (Chard et al. 1991). Disability performing everyday activities was observed in 30% of patients over 65 years (Chakravarty and Webley 1993). Only 50% of people seeking primary care for a first episode of shoulder pain show complete recovery within 6 months and 65% within 1 year (Van der Windt et al. 1996). The risk of a poor long-term outcome due to persistent pain has been observed for conservative or surgical treatments and such patients account for 80% of the total expenses for shoulder pain (Kuijpers et al. 2006). Physical therapy focuses mainly on improving joint motion rather than pain relief or quality of life (Page et al. 2015). Occupational therapy interventions can improve function and decrease pain in shoulder disorders, with the strongest evidence for exercise interventions (Marik and Roll 2017). According to French guidelines for chronic shoulder pain without instability (HAS - Haute Autorité en Santé 2005), the pharmacological approach is based on pain killers, non-steroidal-anti-inflammatory topical agent and corticosteroid injections. As there is no real consensus on the therapeutic management of common shoulder disorders (Marik and Roll 2017), assessment of the effectiveness of treatments is needed (Bot et al. 2004).

Balneotherapy and/or spa therapy (Gutenbrunner et al. 2010) is commonly used in Europe and worldwide for rheumatic conditions (Forestier et al. 2017; Karagülle et al. 2017) such as low back pain (Karagülle and Karagülle 2015), knee osteoarthritis (Forestier et al. 2010; Tenti et al. 2015), hand osteoarthritis (Fioravanti et al. 2014) and fibromyalgia (Bazzichi et al. 2013; Naumann and Sadaghiani 2014). Regarding chronic shoulder pain, a recent pilot randomised controlled trial (Tefner et al. 2015) compared balneotherapy to physiotherapy (exercise and transcutaneous nerve stimulation—TENS) showing a greater improvement in pain and function in the balneotherapy group.

The purpose of this multicentre prospective randomised controlled trial was to assess, in a pragmatic way, using the immediate versus delayed treatment paradigm (Guillemin et al. 1994; Constant et al. 1998), the efficacy of spa therapy combining several spa modalities together with supervised mobilisation in a thermal pool in a population of patients with chronic shoulder pain without instability due to miscellaneous chronic conditions.

The main endpoint was the benefit on pain and disability assessed using a self-reported outcome, the disability arm shoulder hand score (DASH) (Hudak et al. 1996; Beaton et al. 2001), in its short version (Beaton et al. 2005), French validated, the French Quick-DASH (F-QD) (Fayad et al. 2008a, b).

Secondary endpoints were (1) to measure the modifications in quality of life assessed by the French validated version of the Short-Form 36 (SF-36) (Ware and Sherbourne 1992; Pernegger et al. 1995; Leplege et al. 1998); (2) to calculate the effect size (ES) (Kazis et al. 1989) of the improvement in pain and disability, along with the ratio of patients achieving a minimum clinical important improvement (MCII) of the DASH score (improvement of at least 10 points); (3) to assess the impact on the consumption of health products and (4) to evaluate the tolerance of the treatment by recording all adverse events.

## Material and methods

### Patients

Patients with chronic shoulder pain due to tendinitis, bursitis (calcified or not), rotator cuff syndrome or omarthrosis were included in the study. The patients were recruited locally to the thermal therapy facilities in order to be treated on a daily basis and continued to live at home. Information about the trial was given in the local media (newspapers, local TV, radio), and posters were in displayed in local pharmacies and general practitioners’ waiting rooms. All patients were required to give written informed consent.

### Inclusion criteria

Included patients had to be between 20 and 80 years and have chronic shoulder pain (pain duration of more than 6 months). When shoulder pain could be related to a traumatic event, the trauma must have happened at least 6 months previously. Corticosteroid treatment for concomitant disease could be continued at a stable dose. According to the French National Agency for Health Policy (Haute Autorité en Santé) recommendations (HAS - Haute Autorité en Santé 2005), the diagnosis was based on (i) clinical history; (ii) clinical examination of the shoulder: (a) observation, (b) palpation, (c) active and passive range of motion, (d) provoked pain by passive and active movement, muscular testing, rotator cuff and shoulder instability tests, (e) neurological and (f) vascular examinations; (iii) general clinical examination and (iv) X-ray examination (within the previous 18 months).

Clinical examination of the upper limb and neck was carried out according to the European SALTSA consensus (Slutter et al. 2001); an X-ray examination was made with the following views: antero-posterior views in three arm rotations, lateral view of the acromio-clavicular space, visualisation of the acromioclavicular joint, antero-posterior view during resisted abduction of the affected shoulder and/or strict antero-posterior straight-beam decubitus view.

Diagnosis of omarthrosis and calcified tendinitis was based on clinical and contributive X-ray examinations; rotator cuff syndrome with impingement was diagnosed on the clinical symptoms and X-ray examination showing a narrowing of the acromio-humeral distance ± the spur of the acromion, a sclerosed acromion, cystic changes around greater tuberosity, rounded tuberosities and an inferior gleno-humeral spur; the diagnosis of chronic tendinitis (rotator cuff and/or biceps) was based on the clinical data and the lack of significant X-ray modifications (Cotty et al. 1988; Railhac et al. 2001; Saupe et al. 2006).

Nearly every patient had an ultrasonographic examination and some a magnetic resonance imaging (MRI) examination. However, as ultrasonographic examinations were performed by several radiologists (and are difficult to interpret by a doctor who did not perform the examination), and MRI were not performed systematically, according to HAS recommendations, in patients with non-surgical treatment, we decided not to report the data provided by these last two investigations when performed. The various diagnoses are reported in Table 1. Nevertheless, the aim of the study was to assess the symptomatic results of spa therapy on shoulder pain and disability in everyday life. Thus, all patients enrolled in the trial were considered together as “chronic shoulder pain” patients with no further distinction paid to the anatomo-clinical condition.

**Table 1:** Characteristics of patients at baseline

Patient characteristics		W0 (*N* = 185)		*p*
		A (spa therapy) (*n* = 92)	B (controls) (*n* = 93)	
Sex (% women)		52.2	52.1	ns
Age (years) [mean (SD)]		57 (9.9)	58 (9.5)	ns
Body mass index [mean (SD)]		26.5 (4.9)	27.9 (5.2)	ns
Disease duration (months) [mean (SD)]		74.7 (83.3)	79.7 (72.8)	ns
Out of work (%)		56 (60.8)	57 (61.2)	ns
Risk factors for shoulder disorder				
Professional activity, *n* (%)		52 (56.5)	53 (56.9)	ns
Physical activity, *n* (%)		44 (47.8)	36 (38.7)	ns
Shoulder disorder aetiology				
Trauma history, *n* (%)		19 (20.6)	17 (18.3)	ns
Non-traumatic, *n* (%)		73 (79.3)	76 (81.7)	ns
Diagnosis of shoulder disorders				
Chronic tendinopathy (%)		38 (41.3)	44 (47.3)	ns
Calcified tendinopathy (%)		26 (28.2)	23 (24.7)	ns
Rotator cuff impingement (%)		14 (15.2)	15 (16.1)	ns
Omarthrosis (%)		14 (15.2)	11 (11.8)	ns
Treatment				
None (%)		34.8	44.1	ns
Analgesics (%)		38.0	32.3	ns
NSAIDs (%)		7.6	9.7	ns
Both (%)		19.6	14.0	ns
Massage	Users (%)	24.4	24.4	ns
	Mean number (SD)	5.4 (13.8)	3.7 (8.1)	
Rehabilitation	Users (%)	28.1	20.7	ns
	Mean number (SD)	5.5 (11.1)	4.4 (11.2)	
Past steroid injection	Users (%)	20.5	13.0	ns
Topical agent use	Users (%)	70.1	44.6	< 0.001

*SD* standard deviation, *ns* not significant, independent sample *t* test or chi^2^ (*α* = 0.05)

### Exclusion criteria

Patients with the following conditions were not included in the trial: instability of the shoulder, adhesive capsulitis, chronic inflammatory rheumatisms, shoulder pain related to neurological, vascular or cancer, secondary shoulder pain due to visceral conditions, inability to complete questionnaires because of cognitive impairment or language difficulty. Contraindications were as follows: immune deficiency, evolving cardiovascular conditions, cancer, infection, non-equilibrated diabetes mellitus or intolerance to any aspect of spa treatment; spa treatment within the previous 6 months; steroid injection within the previous 3 months; physiotherapy in the previous month; changes in non-steroidal anti-inflammatory drug (NSAID) administration within the previous 5 days or in other analgesic drug use within the previous 12 h.

### Intervention

Patients randomly assigned to the treatment group at inclusion (group A) received immediate spa therapy consisting of 18 days of daily treatment for 3 weeks, 6 consecutive days a week. Patients randomly assigned to control group received the same spa treatment 6 months later (group B) in an immediate versus delayed treatment paradigm. According to symptomatic needs, the patients could continue to use pain-killers and/or topical agents and/or perform physical therapy prescribed by their primary care physician during the 6-month follow-up period. In France, spa treatments are approved and controlled by the ‘General Direction of Health’ under the Ministry of Health. The standardised shoulder spa therapy program was designed by experienced physicians from the different spa therapy facilities participating to the trial in accordance with national recommendations for practices in thermal centres (Syndicat national des médecins thermaux 2004).

Spa treatment included the following: bubbling baths with spa mineral water at 36 °C for 15 min, direct applications of hydro-mineral mud at 45 °C to the shoulder for 20 min, hydrojet sessions with spa mineral water at 39 °C for 7 min and collective general mobilisation sessions (passive mobilisation with gradual active self-mobilisation and introduction of active joint exercises below the pain threshold) in a collective mineral water pool at 32 °C for 20 min supervised by a registered physiotherapist. Attendance, tolerance and proper performance of the various treatments were monitored by an independent trained physician from the spa centre at the beginning, middle and end of the 3-week treatment period.

During follow-up, patients requiring surgery, morphinic drugs, local steroid injections or doses of systemic steroid treatments were withdrawn from the study. All other treatments were authorised and self-recorded by the patients.

This prospective multicentre randomised controlled trial was carried out in three balneotherapy centres in northeastern France (Amnéville les Thermes, Bourbonne les Bains, Plombières) and in one centre in Luxemburg (Mondorf les Bains). These resorts have spa therapy facilities and have extensive experience treating rheumatic patients, as shown in Table 2. The large number of rheumatic patients meant that our study patients were treated as usual patients. The characteristics of the different natural mineral waters in these spa centres are reported in Table 3. The muds were not peloids (Gomes et al. 2013) but were prepared extemporaneously by mixing a clay provided by external suppliers [type smectite: Si_4_0_10_Al_5/3_Mg_1/3_Na_1/3_(OH)_2_] with the natural mineral water of the centre (two parts clay with one part water).

**Table 2:** Details for the four spa therapy centres: number of patients treated annually (total and those with rheumatic conditions) (courtesy CNETH: Conseil National des Etablissements thermaux, Paris, France—http://www.medecinethermale.fr) and patients enrolled by this study

Spa therapy centre	All patients	Rheumatology patients	Inclusions	Follow-up		
	(*N*/year)	(*N*/year)	(*N*)	(*N*)		
			W0	W3	M3	M6
Amnéville	14,795	13,654	87	87	81	78
Bourbonne	8069	7555	79	78	73	67
Plombières	3918	2768	10	9	6	5
Mondorf	4375	3225	9	9	9	9
Total	31,157	27,202	185	184	169	159

**Table 3:** Centres: main features of the spa mineral water

Centres	Amnéville	Bourbonne	Mondorf	Plombière
Water				
Mineral content (mg/l)				
Total	15,200	7484	14,535	306
Na++	3710	2000	3420	76
Ca++	1600	460.5	1680	8.1
Mg++	92.8	14.58	116	1
K+	124	160	120	4.2
Fe	16	160	4.3	< 5
Mn−−	0.49	0.248	0.49	< 1
CO3H-	127	106.3	134	95
SO4−−	1390	982.3	1150	68.4
Cl−	8100	3431.7	7732	6
*T* (°C)	40.3	62.9	12	56
Conductivity (μS/cm)	23,000	11,290	21,832	400
pH	6.81	7.55	6.9	8

### Assessment

At baseline (W0) (T0), before randomisation, a physician recorded demographic and clinical data: age, sex, body mass index, disease duration and treatment use. Both groups were assessed at the end of spa therapy (group A) or at 3 weeks (group B) (W3) (T1); 3 months (M3) after spa therapy or at 15 weeks for controls (T2); 6 months (M6) after spa therapy or at 27 weeks for controls (T3) by a physician blind to the randomisation arm, who completed the case report form and entered the self-assessment questionnaire answers filled-in by the patients. Outcome measures were collected at each visit and the same questionnaires completed. Patients filled-in the F-QD and the SF-36 questionnaires. The F-QD explores (1) physical activity involving the arm, shoulder or hand (6 items); (2) severity of pain and tingling (2 items) and (3) social activities, work and sleep (3 items). Each item has five response options, ranging from 1, ‘no difficulty or no symptom,’ to 5, ‘unable to perform activity or very severe symptom’. A score ranging from 0 (no disability) to 100 (most severe disability) was then calculated (Beaton et al. 2001). Data from patients with more than one unanswered item on the questionnaire were excluded. The time for completing the different questionnaires was about 20 min at each assessment visit. All adverse events were recorded throughout the 6-month follow-up period.

### Sample size determination

The sample size was determined on the basis of a preliminary open study with 36 consecutive patients fulfilling inclusion criteria for whom the mean F-QD score was 48.10 ± 14.24. An improvement of 20% was hypothesized, i.e. a diminution of 10 points (minimal detectable change—MDC) for the F-QD score in the spa therapy group (Roy et al. 2009). Using a predefined α-risk at 5% and ß-risk at 10%, the minimum number of patients needed was calculated at 53 per group or 70 allowing for 20% loss to follow-up. Thus, 140 patients needed to be enrolled in the trial.

### Randomisation

Eligible patients were randomly assigned to the immediate spa therapy or to the control groups using a centralised computer site. Randomisation was stratified by centre and made up of blocks of six with random order. Concealment was assumed by protection of the computer file. The study protocol was approved by the regional ethics committee (CPP: 08-12-0, Nancy, France), the French National Authorities (Afssaps: 2008-A00786-49) and registered on www.clinicaltrials.gov (NCT 01692249).

### Statistical analyses

Data were analysed in intention to treat (ITT) by a statistician, blinded to the randomisation arm, using SPSS 13.0 for Windows (version 13.0; SPSS Inc., Chicago, IL) and checked for missing values and normality. Demographic and clinical data were expressed as mean with standard deviation, median and range. Differences in baseline characteristics and baseline values of the outcome measures between the spa therapy group and controls were tested with an independent sample *t* test (*α* = 0.05). In the event of significant differences between the two groups in baseline characteristics, adjustments were made in statistical analysis. The main endpoint was assessed at 6 months and was the mean change in the F-QD between baseline (WO) and the follow-up visit (M6). The effect size (ES) for spa therapy was then calculated from the spa group F-QD at 6 months. Secondary endpoints were the mean change in the F-QD at 3 weeks (W3) and 3 months (M3); the proportion of patients with a minimal clinically important improvement (MCII) (change in DASH > 10 points), at each time point (Roy et al. 2009), quality of life (SF-36) and medication consumption at each time point. Mean changes in the spa therapy and control groups were compared using Student’s *t* test. Multivariate covariance analysis was conducted adjusted on age, sex and initial F-QD and SF-36 scores. Qualitative variables were compared using a chi-squared test or Fisher test. The statistical analysis of the data was carried out in ‘intention to treat’ (ITT).

## Results

### Descriptive analysis

**Fig. 1:**
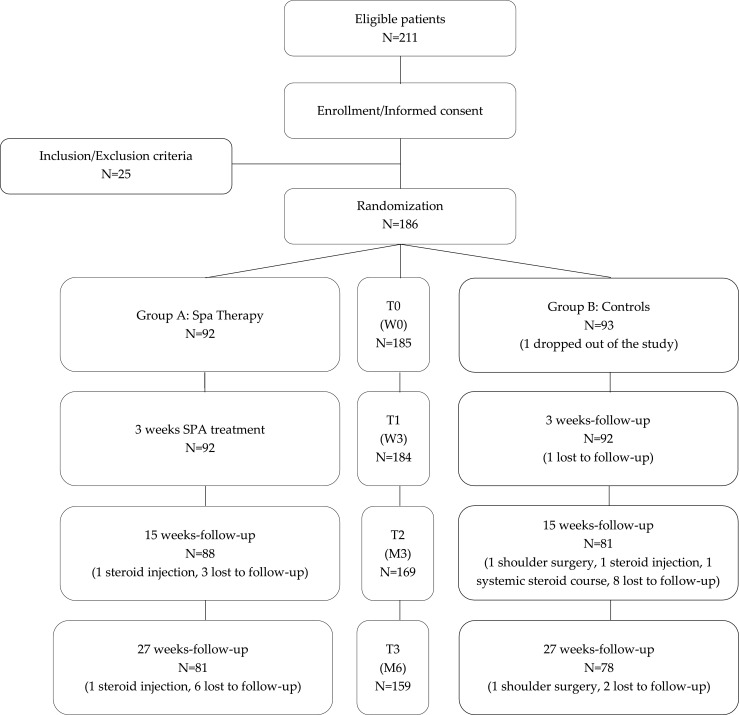
Study flow chart

The overall flow chart of patients included during the 7-month period of enrolment is shown in Fig. 1. At baseline, 186 subjects were randomised: 92 to group A (immediate spa therapy) and 94 to group B (control subjects with spa therapy delayed by 6 months). The baseline characteristics of patients are summarised in Table 3. Mean age of patients was 57.9 ± 9.7 years (range 21–81), and 52% were female. There were no statistically significant differences between the groups in age, sex, BMI, disease duration, actual or past risk factors for shoulder disorders (professional or physical activity) or being out of work and for diagnosis of shoulder disorders. Despite randomisation, the mean F-QD was higher in spa therapy group (48.5 ± 11.9) in comparison to the control group (41.7 ± 16.8) (*p* < 0.01) (Table 4). However, this difference did not reach MDC. Differences between groups were also observed at baseline for two components of the SF-36: physical functioning and physical condition (respectively, 21.5 ± 3.9 versus 23.2 ± 4.2 (*p* = 0.004) and 4.8 ± 1.2 versus 5.4 ± 1.5 (*p* = 0.013) in group A versus group B). Concerning treatments, no statistically significant difference was observed between groups except for topical agents being more frequently used in the spa therapy group (Table 5).

**Table 4:** Changes in mean F-QuickDASH and SF-36 components

	W3-W0		*p*	M3-W0		*p*	M6-W0		*p*
Group	A	B		A	B		A	B	
*N*	(92)	(92)		(88)	(81)		(81)	(78)	
F-QuickDASH (mean)	− 12.0	0.5	< 0.001	− 19.0	1.5	< 0.001	− 15.8	3.4	< 0.001
SF—36 (mean)									
Physical functioning	9.0	0.5	0.012	18.5	− 0.1	< 0.001	15.5	− 0.1	< 0.001
Physical condition	13.4	− 2.2	0,007	22.3	− 5.7	< 0.001	12.1	− 4.5	0.015
Bodily pain	10.1	− 0.1	< 0.001	20.1	− 2.2	< 0.001	15.9	0.7	< 0.001
General health	3.2	− 2.0	0.021	4.2	− 5.7	< 0.001	4.6	− 5.7	< 0.001
Vitality	− 0.9	− 0.5	ns	5.7	− 1.1	0.027	3.6	− 2.3	ns
Social functioning	6.5	2.4	ns	10.6	− 2.3	< 0.001	6.3	− 2.1	0.049
Emotional state	6.6	− 2.5	ns	5.4	1.6	0.609	1.6	− 1.7	ns
Mental health	8.2	3.4	ns	5.2	1.6	ns	4.3	− 1.4	ns

*Group A* immediate spa treatment, *group B* delayed spa treatment, **i.e.** controls; *DASH* disability of arm, shoulder and hand; *SF-36* short form 36 questionnaire, *NSAIDs* non-steroidal anti-inflammatory drugs, *ns* not significant, *t* test or chi^2^)

**Table 5:** Changes in treatment use over time

	W3-W0		*p*	M3-W0		*p*	M6-W0		*p*
Group	A	B		A	B		ST	C	
*N*	(92)	(92)		(88)	(81)		(81)	(78)	
Treatment use (%)									
None	2.6	− 16.4	0.046	3.8	− 6.3	ns	− 9.8	− 21.1	ns
Analgesics	1.6	10.3	0.046	0.6	− 3.6	ns	− 3.0	0.7	ns
NSAIDs	− 1.0	0.9	0.046	− 4.2	0.6	ns	− 3.6	− 2.7	ns
Both	− 3.1	5.1	0.046	− 0.3	5.3	ns	0.4	3.0	ns
Massage	− 13.4	− 13.8	0.012	− 12.9	− 4.9	ns	− 10.1	− 18.1	ns
Rehabilitation	− 19.3	− 7.9	ns	− 18.8	− 10.4	ns	− 23.3	− 13.1	ns
Topical agents	− 47.0	− 8.4	< 0.001	− 37.8	− 1.3	0.059	− 50.9	− 15	0.003

*Group A* immediate spa treatment, *group B* delayed spa treatment, **i.e.** controls; *NSAIDs* non-steroidal anti-inflammatory drugs, *ns* not significant, *t* test or chi^2^

### Comparative analysis

#### Main endpoint and changes in F-QuickDASH

**Fig. 2:**
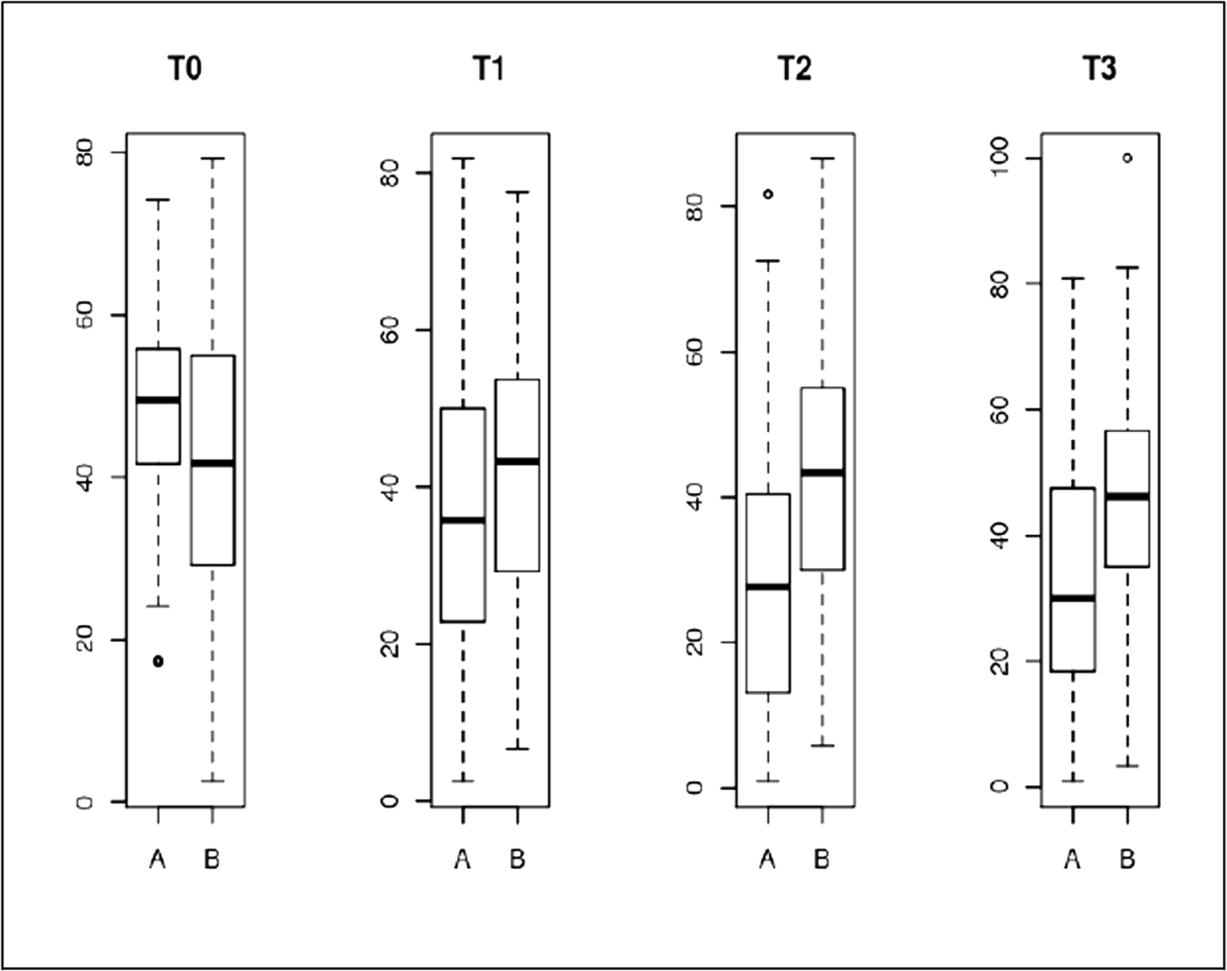
F-QuickDASH follow-up in both groups (A: spa therapy, B: controls) at W0 (T0), W3 (T1), M3 (T2) and M6 (T3)

At M6 (T3), 20 patients (10.7%) were lost to follow-up: nine in group A and 11 in group B; six patients were withdrawn from the study (3.2%): two in group A and four in group B (p NS). The baseline characteristics of the patients withdrawn from the study and the completers showed no significant differences. Changes in the F-QD over time are summarised in Table 4 and Fig. 2. The mean changes of the primary endpoint (F-QD) at M6 were − 15.8 points (− 32.6%) in group A and + 3.4 points (+ 8.15%) in group B (*p* < 0.001). The number of patients with a MCII at the different assessment times was significantly higher in the spa therapy group compared to controls (53.3 versus 14.1% at W3, 67.8 versus 12.3% at M3 and 59.3 versus 17.9% at M6). The effect size for spa therapy based on F-QD was calculated at 1.32 (95%CI: 1.68–0.97). Although comparative analysis was planned on the mean changes in F-QD between groups and not on direct comparison, the baseline difference was accounted for in the multivariate analysis. Multivariate analysis showed that (i) the changes in F-QD at M6 were not related to baseline characteristics of patients in age, sex, BMI or disease duration and (ii) the mean difference in F-QD changes significantly favoured spa therapy at each time point (*p* < 0.001).

#### Secondary endpoints

*Changes in SF-36* components over time are presented in Table 4. At 6 months, the difference in mean changes in scores was statistically significant between the groups with greater improvement in the spa therapy group for five components: physical functioning, physical condition, body pain, general health and social functioning. The baseline differences were also considered in the multivariate analysis. Thus, the difference in changes between groups remained statistically significant in multivariate analysis for all five components in favour of spa therapy. Furthermore, physical functioning appeared significantly related to sex and BMI (*p* = 0.01) and social functioning to age (*p* = 0.01).

*Changes in treatment use* over time are presented in Table 5. The use of analgesics and/or oral NSAIDs (*p* = 0.046) and of massages (*p* = 0.012) decreased significantly during spa therapy but not after 3 months. The use of topical agents was significantly reduced in the spa therapy group at 3 months (− 47 versus − 8.4%; *p* < 0.001) and remained significantly lower at 6 months (− 23.3 versus − 13.1%; *p* = 0.003).

#### Adverse events

Adverse events are reported in Table 6; the survey of treatments during the 6-month follow-up period showed a low prevalence of adverse events; 24 events were recorded by patients in the spa therapy group and 29 by patients of the control group; the difference was not significant. Spa therapy appeared to be well tolerated by the patients included in the trial as has been observed for rheumatic conditions (Roques and Queneau 2016).

**Table 6:** Adverse events recorded during follow-up in both groups

Endpoint group	W3		M3		M6		Total		
	A (*N* = 92)	B (*N* = 92)	A (*N* = 88)	B (*N* = 81)	A (*N* = 81)	B (*N* = 78)	A	B	*p*
All adverse events (AE)	8	7	10	15	6	7	24	29	ns
Severe adverse events	0	0	3	1	1	1	4	2	ns
AE with withdrawal from the study	0	1	2	3	1	0	3	4	
Shoulder surgery or CT treatment	0	0	2	2	1	1	3	3	
Infections	3	3	2	4	0	1	5	8	
Rheumatologic disorders	3	1	6	8	3	3	12	12	

*A* spa therapy group, *B* control group, *CT* corticosteroids, *ns* not significant, chi^2^ or Fisher test

## Discussion

This study was a prospective single-blind randomised controlled trial (RCT), with an intention to treat analysis on a population (of 186 patients) whose size was a priori calculated to guarantee an accurate control of the alpha and beta risks. To date, it is the first investigation on chronic shoulder pain using a RCT methodology of the effectiveness of spa therapy combining hydrothermal therapy along with supervised exercise in a mineral water pool. It showed an improvement in pain and disability using the DASH score, in its F-QD version, and in quality of life assessed by the SF-36. Only one other randomised study addressing the same condition was found in the international literature. This study, by Tefner et al. (2015), included 49 patients with shoulder disorders treated by balneotherapy, showed an improvement of pain and disability but not of quality of life respectively assessed by the Shoulder Pain and Disability Index (SPADI) (Roach et al. 1991) and the SF36. That pilot trial compared 15 sessions (over 4 weeks) of balneotherapy plus physiotherapy to physiotherapy alone. In our study, the patients benefited from 18 sessions of spa therapy and supervised self-mobilisation in a mineral water pool over 3 weeks. The spa therapy was the usual spa therapy delivered in France and reimbursed by the social security; it combines several hydro-thermal treatments with rehabilitation treatments using mineral water for massages and exercise.

The 11 items of the validated Quick DASH have the same metrology qualities as the 30-item DASH (Beaton et al. 2001), regarded as the international reference for self-assessment of disability of an upper limb (Bot et al. 2004; Roy et al. 2009; Staples et al. 2010); the Quick DASH is easier to administer and to exploit. The validity and reliability of the F-QD (Fayad et al. 2008a, b) have been established for degenerative and traumatic disorders of the shoulder (Fayad et al. 2008a, b). The baseline values of F-QD in that trial’s population ranged from 40 to 50/100, showing a good concordance with the preliminary data recorded. DASH score values ranged from 3 to 6/100 in healthy people and reached 28 ± 25 in people with psoriatic arthritis, 77 ± 22 in patients with frozen shoulder (Navsarikar et al. 1999; Griggs et al. 2000; Wolf and Green 2002). The disability due to shoulder impairment in the study’s population was severe and similar to the disability of patients requiring total shoulder replacement (TSR) (Skutek et al. 2000; Deshmukh et al. 2005; Angst et al. 2008).

The calculated ES for pain and disability was 1.32, a high level of improvement as the ES for total shoulder replacement ranged from 0.70 in 109 patients with miscellaneous shoulder conditions (Gummesson et al. 2003) to 1.19 in 162 patients with rheumatoid or degenerative shoulder impairment (Angst et al. 2008).

Pain, disability and range of motion are common endpoints in clinical trials for shoulder disorders but the patient’s health perception is crucial for both patient’s self-assessment and treatment (Staples et al. 2010); therefore, the assessment of QOL is relevant in patients with shoulder disorders. The SF-36 is the most used generic instrument for assessing QOL, with physical, mental and psychosocial dimensions. It showed at M6 a significant improvement in the dimensions of body pain, physical condition and functioning, general health and social functioning. Vitality, emotional state and mental functioning were not modified as shoulder disorders are essentially a physical problem. Changes in SF-36 were concordant with the efficacy of spa therapy evaluated by the F-QD score. The absence of improvement of quality of life in the study by Tefner et al. (2015) could be related to their limited population and to the use of the SF36 which is more appropriate to lower limb impairments (climbing stairs, walking distance, bending, etc.) possibly leading to a lower response than shoulder specific assessment tools.

A reduction in consumption of topical agents was observed in line with the local improvement. The consumption of analgesics and NSAIDs was found unchanged, but this outcome was recorded as a binary parameter preventing the identification of possible reduced consumption.

Spa therapy was well tolerated. No unexpected adverse events were reported. The serious adverse events recorded were shoulder surgery for worsening of the initial condition (two patients in each group).

Exercise improves pain and function in patients with shoulder impingement (Van der Windt et al. 1995), and its role in our trial needs to be discussed. However, the self-mobilisation performed by our patients cannot be compared to conventional rehabilitation made up of 30- to 40-min sessions of exercises, two to five times a week, for 6 weeks to 5 months (Gleyze et al. 2011). It is hard to settle the case as shoulder disorders are quite heterogeneous, the physical treatments can be different and the methodological quality of trials in the field are variable, illustrated by the discordant conclusions of reviews (Hanratty et al. 2012; Ellenbecker and Cools 2010; Shire et al. 2017). A systematic review of physical therapy trials (Page et al. 2015) reported that most of the 171 selected trials assessed mainly pain (87%), function (72%) and range of motion (67%); adverse events, overall assessment of treatment success and health-related QOL was considered in only 18–27% of trials, and work disability and referral for surgery were determined in less than 5% of trials. Numerous measurement instruments (35 for pain and 29 for function) have been used; the primary endpoint was specified in only 27% of cases (mainly pain and degree of impingement syndrome and range of motion in adhesive capsulitis).

Methodological issues should be discussed. This large multicentre trial suggests important clinical results observed with ITT analysis using a robust assessment tool (F-QD). Furthermore, the patients attended the sessions in the spa therapy facilities whilst continuing to live at home; so, the trial assessed the spa treatment alone outside the role of a 3-week stay in a resort and the modifications in daily habits it produces; this particular situation has been previously used in large trials (Forestier et al. 2010; Carpentier et al. 2014).

The concealment of the randomisation arm from investigators was easy to maintain as the main clinical assessment was prior to randomisation, and assessment at the other times was made using patients’ self-reported outcomes; the physicians completing the CRF were blinded to the randomisation, and the patients were asked not to reveal the group they belonged to. The randomisation arm was easy to conceal from the statisticians.

The treatment was highly standardised reducing any eventual differences between the centres, and the mineral content of the waters was quite similar for three out of the four centres (Table 3). The water at the fourth centre differed but, due to the relatively small number of patients treated in this facility, its influence on the overall results remains minor. A subgroup analysis by centre would have needed a much larger number of patients which was not feasible and of questionable relevance.

Some limitations should be addressed. The ‘immediate versus delayed treatment’ paradigm could be considered as producing a dissatisfaction bias due to the 6-month wait before spa therapy for control patients treated initially by usual care. The large number of patients enrolled and the lower number of patients lost to follow-up than expected do not support the idea of dissatisfaction. The paradigm we used allowed randomisation after clinical assessment favouring the constitution of a reliable control group. The unexpected differences between the two groups, observed in F-QD baseline scores before randomisation and not explained by demographical or clinical differences, remain unexplained and in addition do not support the existence of a bias of dissatisfaction.

The results have been analysed without paying attention to the anatomo-nosological classification, as did also Tefner et al. (2015). The frequent fluctuations and association of periarticular lesions and the ‘real-life practice’ assessed by this trial make our approach relevant. The demonstration of the medical benefit of spa therapy, needed by health authorities (French National Agency for Health Policy) and reimbursement schemes, makes our pragmatic approach for this conservative treatment highly relevant. In contrast, an assessment of a surgical treatment would have needed an accurate description of the anatomical lesions. An explanative approach would have required us to consider the results according to the anatomical lesions, but the significance, the feasibility and the cost of such a trial would have been very different.

## Conclusion

In this particular population, spa therapy consisting of mineral water baths and mud applications together with supervised self-mobilisation in a thermal pool provided a statistically significant benefit in pain, function, and quality of life in patients with chronic shoulder pain after 6 months compared with usual care. In order to optimise and rationalise the best use of spa therapy, these results need to be completed by other studies that better discriminate between the different conditions that make up of chronic shoulder disorders.
